# A new bound for the spectral radius of nonnegative tensors

**DOI:** 10.1186/s13660-017-1362-7

**Published:** 2017-04-26

**Authors:** Suhua Li, Chaoqian Li, Yaotang Li

**Affiliations:** School of Mathematics and Statistics, Yunnan University, Kunming, Yunnan 650091 P.R. China

**Keywords:** nonnegative tensor, weakly irreducible, spectral radius, Perron eigenpair

## Abstract

By estimating the ratio of the smallest component and the largest component of a Perron vector, we provide a new bound for the spectral radius of a nonnegative tensor. And it is proved that the proposed result improves the bound in (Li and Ng in Numer. Math. 130(2):315-335, 2015).

## Introduction

Let $\mathbb{C}$ ($\mathbb{R}$) be the set of all complex (real) numbers, $\mathbb{R}_{+}$ ($\mathbb{R}_{++}$) be the set of all nonnegative (positive) numbers, $\mathbb{C}^{n}$ ($\mathbb{R}^{n}$) be the set of all dimension *n* complex (real) vectors, and $\mathbb {R}_{+}^{n}$ ($\mathbb{R}_{++}^{n}$) be the set of all dimension *n* nonnegative (positive) vectors. An order *m* dimension *n* complex (real) tensor $\mathcal{A}=(a_{i_{1}i_{2}\cdots i_{m}})$, denoted by $\mathcal{A} \in\mathbb{C}^{[m,n]}$ ($\mathcal{A}\in\mathbb {R}^{[m,n]}$, respectively), consists of $n^{m}$ entries:

$$a_{i_{1}i_{2}\cdots i_{m}}\in\mathbb{C}\ (\mathbb{R}),\quad \forall i_{k}\in N= \{1,2,\ldots,n\}, k=1,2,\ldots,m. $$

A tensor $\mathcal{A}=(a_{i_{1}i_{2}\cdots i_{m}})\in\mathbb{R}^{[m,n]}$ is called nonnegative if

$$a_{i_{1}i_{2}\cdots i_{m}}\geq0,\quad \forall i_{k}\in N, k=1,2,\ldots,m. $$

As the eigenvalues of matrices have many extensive applications, the *H*-eigenvalues [2] for higher order tensors also have a wide range of applications such as numerical multilinear algebra and higher order Markov chains [3–5].

### Definition 1

[2]

Let $\mathcal{A}=(a_{i_{1}i_{2}\cdots i_{m}})\in\mathbb{C}^{[m,n]}$. Then $(\lambda, x)\in\mathbb{C}\times\mathbb{C}^{n}\backslash\{0\}$ is called an eigenpair of $\mathcal{A}$ if

$$\mathcal{A}x^{m-1}=\lambda x^{[m-1]}, $$

where $\mathcal{A}x^{m-1}$ and $x^{[m-1]}$ are dimension *n* vectors with *i*th entries

$$\bigl(\mathcal{A}x^{m-1}\bigr)_{i}=\sum _{i_{2},\ldots,i_{m}\in N}a_{ii_{2}\cdots i_{m}}x_{i_{2}}\cdots x_{i_{m}}, $$

and

$$x^{[m-1]}_{i}=x_{i}^{m-1}. $$

Specifically, $(\lambda, x)$ is called an *H*-eigenpair if $(\lambda, x)\in\mathbb{R}\times\mathbb{R}^{n}$.

Recently, the spectral radius problem for nonnegative tensors has attracted special attention of researchers [6–9]. In [6], Chang et al. generalized the famous Perron-Frobenius theorem [10] for irreducible nonnegative matrices to irreducible nonnegative tensors.

### Definition 2

[6]

Let $\mathcal{A}=(a_{i_{1}i_{2}\cdots i_{m}})\in\mathbb{C}^{[m,n]}$. $\mathcal{A}$ is called reducible if there is a nonempty proper subset $K\subset N$ such that

$$a_{i_{1}i_{2}\cdots i_{m}}=0,\quad \forall i_{1}\in K, \forall i_{2},\ldots, i_{m}\notin K. $$

If $\mathcal{A}$ is not reducible, then we call $\mathcal{A}$ irreducible.

### Theorem 1

[6]

*Let* $\mathcal{A}=(a_{i_{1}i_{2}\cdots i_{m}})\in\mathbb{R}^{[m,n]}$ *be an irreducible nonnegative tensor and*

$$\rho(\mathcal{A})=\max\bigl\{ \vert \lambda \vert : \lambda\in\sigma( \mathcal{A})\bigr\} $$

*be the spectral radius of* $\mathcal{A}$, *where*

$$\sigma(\mathcal{A})=\{\lambda: \lambda\textit{ is an eigenvalue of }\mathcal {A} \}. $$

*Then* $\rho(\mathcal{A})> 0$ *is an eigenvalue of* $\mathcal{A}$ *with a positive eigenvector* *x* *corresponding to it*.

Note that $\rho(\mathcal{A})$ and *x* in Theorem 1 are called the Perron root and the Perron vector of $\mathcal{A}$, respectively, and $(\rho(\mathcal{A}), x)$ is regarded as a Perron eigenpair.

Subsequently, Friedland et al. generalized the result in Theorem 1 to weakly irreducible [7] nonnegative tensors in [8].

### Definition 3

[7]

Let $\mathcal{A}=(a_{i_{1}i_{2}\cdots i_{m}})\in\mathbb{C}^{[m,n]}$, define a matrix $M=(M_{ij})\in\mathbb{C}^{n\times n}$ with

$$M_{ij}=\sum_{j\in\{i_{2},\ldots,i_{m}\}}|a_{ii_{2}\cdots i_{m}}|,\quad \forall i\in N, \forall j\in N. $$

$\mathcal{A}$ is called weakly irreducible if *M* is an irreducible matrix.

### Theorem 2

[8]

*Let* $\mathcal{A}=(a_{i_{1}i_{2}\cdots i_{m}})\in\mathbb{R}^{[m,n]}$ *be a weakly irreducible nonnegative tensor*. *Then* $\rho(\mathcal{A})> 0$ *is an eigenvalue of* $\mathcal{A}$ *with a positive eigenvector* *x* *corresponding to it*.

In [9], Yang and Yang generalized the result in Theorem 1 to nonnegative tensors.

### Theorem 3

[9]

*Let* $\mathcal{A}=(a_{i_{1}i_{2}\cdots i_{m}})\in\mathbb{R}^{[m,n]}$ *be a nonnegative tensor*, *then* $\rho(\mathcal{A})\geq0$ *is an eigenvalue of* $\mathcal{A}$ *with a nonnegative eigenvector* *x* *corresponding to it*.

For the spectral radius of a nonnegative tensor $\mathcal{A}$, although some algorithms of calculating its value were proposed [11–13], it is not easy to choose an appropriate iterative initial value such that these iterative methods rapidly converge to its exact value. Therefore, it is necessary to give an initial estimate for the spectral radius of a nonnegative tensor. Actually, there are already some results for the bound of the nonnegative tensors’ spectral radius, for example, Yang and Yang extended the classical spectral radius bound for nonnegative matrices to nonnegative tensors in [9] and obtained the following result.

### Theorem 4

[9]

*Let* $\mathcal{A}=(a_{i_{1}i_{2}\cdots i_{m}})\in\mathbb{R}^{[m,n]}$ *be a nonnegative tensor*, *then*

1 $$\begin{aligned} r\leq\rho(\mathcal{A})\leq R, \end{aligned}$$

*where*

$$ r_{i}(\mathcal{A})=\sum_{i_{2},\ldots,i_{m}\in N}a_{ii_{2}\cdots i_{m}}, \qquad r=\min_{i\in N}r_{i}(\mathcal{A}),\qquad R=\max _{i\in N}r_{i}(\mathcal{A}). $$

In [1], by estimating the ratio of the smallest component and the largest component of a Perron vector, Li and Ng gave the following bound for the spectral radius of a nonnegative tensor and proved it is better than the bound in (1).

### Theorem 5

[1]

*Let* $\mathcal{A}=(a_{i_{1}i_{2}\cdots i_{m}})\in\mathbb{R}^{[m,n]}$ *be a nonnegative tensor*, *then*

2 $$ \nu(\mathcal{A})\leq\rho(\mathcal{A})\leq\omega(\mathcal{A}), $$

*where*

$$\begin{aligned}& \nu(\mathcal{A})=\min_{i,j\in N} \biggl\{ a_{ij\cdots j} \biggl( \frac {1}{\tau(\mathcal{A})^{m-1}}-1 \biggr)+r_{i}(\mathcal{A}) \biggr\} , \\& \omega( \mathcal{A})=\max_{i,j\in N} \bigl\{ r_{i}(\mathcal {A})-a_{ij\cdots j}\bigl(1-\tau(\mathcal{A})^{m-1}\bigr) \bigr\} , \\& \tau(\mathcal{A})= \biggl(\frac{r-\beta_{0}(\mathcal{A})}{R-\beta _{0}(\mathcal{A})} \biggr)^{\frac{1}{2(m-1)}}, \\& \beta_{0}(\mathcal {A})=\min_{i,j\in N}\{a_{ij\cdots j} \}. \end{aligned}$$

*Furthermore*, $r\leq\nu(\mathcal{A})\leq\omega(\mathcal{A})\leq R$.

In this paper, we continue to study this problem and present a new lower bound and a new upper bound for the spectral radius of a nonnegative tensor by giving a new ratio of the smallest component and the largest component of a Perron vector. It is proved that this bound is better than the bound in (2). Numerical examples are also given to illustrate the efficiency of the proposed results.

## Bounds for the spectral radius of nonnegative tensors

In this section, we first give a lemma to estimate the ratio of the smallest component and the largest component of a Perron vector, and then we give a bound for the spectral radius of nonnegative tensors.

### Lemma 1

*Let* $\mathcal{A}=(a_{i_{1}i_{2}\cdots i_{m}})\in\mathbb{R}^{[m,n]}$ *be a weakly irreducible nonnegative tensor with a Perron vector* *x*, *and let* $x_{s}=\min_{i\in N}\{x_{i}\}$, $x_{l}=\max_{i\in N}\{x_{i}\}$. *Then*

$$\frac{x_{s}}{x_{l}}\leq\zeta(\mathcal{A}), $$

*where*

$$\begin{aligned}& \zeta(\mathcal{A})= \biggl\{ \frac{r-\beta_{0}(\mathcal{A})-\sum_{k=1}^{m-2} [\binom{m-1}{k}(n-1)^{k}\beta_{k}(\mathcal{A}) (1- (\frac{r-\beta_{0}(\mathcal{A})}{R-\beta_{0}(\mathcal{A})} )^{\frac{m-k-1}{2(m-1)}} ) ] }{R-\beta_{0}(\mathcal{A})} \biggr\} ^{\frac{1}{2(m-1)}}, \\& \beta_{t}(\mathcal{A})=\min_{i,j\in N}\bigl\{ a_{ii_{2}\cdots i_{m}}: (i_{2},\ldots,i_{m})\in\triangle(j; m-t-1)\bigr\} ,\quad t=0,1,\ldots,m-2, \\& \triangle(j; u) =\bigcup_{\substack{S\subseteq\{2,\ldots,m\},\\ |S|=u}}\bigl\{ (i_{2},\ldots,i_{m}): i_{v}=j, \forall v\in S, \textit{ and } i_{v}\neq j, \forall v\notin S\bigr\} ,\quad u=0,1,\ldots,m-1. \end{aligned}$$

### Proof

Since $\mathcal{A}$ is a weakly irreducible nonnegative tensor, according to Theorem 2, we have $(\rho(\mathcal{A}), x)\in \mathbb{R}_{++}\times\mathbb{R}_{++}^{n}$ is a Perron eigenpair of $\mathcal{A}$. Without loss of generality, suppose that $r_{p}(\mathcal {A})=R$, $r_{q}(\mathcal{A})=r$. By $\mathcal{A}x^{m-1}=\rho(\mathcal {A}) x^{[m-1]}$, we have that for each $i\in N$,

3 $$\begin{aligned} \rho(\mathcal{A})x_{i}^{m-1} =&\sum _{i_{2},\ldots,i_{m}\in N}a_{ii_{2}\cdots i_{m}}x_{i_{2}}\cdots x_{i_{m}} \\ =&a_{il\cdots l}x_{l}^{m-1}+\sum _{\substack{(i_{2},\ldots,i_{m})\neq(l,\ldots,l),\\ i_{2},\ldots,i_{m}\in N}}a_{ii_{2}\cdots i_{m}}x_{i_{2}}\cdots x_{i_{m}} \\ \geq& a_{il\cdots l}x_{l}^{m-1}+\sum _{\substack{(i_{2},\ldots,i_{m})\neq (l,\ldots,l),\\ i_{2},\ldots,i_{m}\in N}}a_{ii_{2}\cdots i_{m}}x_{s}^{m-1} \\ =&a_{il\cdots l}\bigl(x_{l}^{m-1}-x_{s}^{m-1} \bigr)+r_{i}(\mathcal{A})x_{s}^{m-1}. \end{aligned}$$

Taking $i=p$ in (3), we obtain that

4 $$ \rho(\mathcal{A}) x_{p}^{m-1}\geq a_{pl\cdots l}\bigl(x_{l}^{m-1}-x_{s}^{m-1} \bigr)+Rx_{s}^{m-1}. $$

Multiplying $x_{p}^{-(m-1)}$ on both sides of (4) gives

5 $$\begin{aligned} \begin{aligned}[b] \rho(\mathcal{A})&\geq a_{pl\cdots l} \biggl(\frac {x_{l}^{m-1}-x_{s}^{m-1}}{x_{p}^{m-1}} \biggr)+R \frac {x_{s}^{m-1}}{x_{p}^{m-1}} \\ &\geq \beta_{0}(\mathcal{A}) \biggl(\frac {x_{l}^{m-1}-x_{s}^{m-1}}{x_{p}^{m-1}} \biggr)+R \frac {x_{s}^{m-1}}{x_{p}^{m-1}} \\ &\geq \beta_{0}(\mathcal{A}) \biggl(\frac {x_{l}^{m-1}-x_{s}^{m-1}}{x_{l}^{m-1}} \biggr)+R \frac {x_{s}^{m-1}}{x_{l}^{m-1}} \\ &=\beta_{0}(\mathcal{A})+\bigl(R-\beta_{0}(\mathcal{A}) \bigr) \biggl(\frac {x_{s}}{x_{l}} \biggr)^{m-1}. \end{aligned} \end{aligned}$$

Similarly, we have that for each $i\in N$,

6 $$\begin{aligned} \rho(\mathcal{A}) x_{i}^{m-1} =&\sum _{i_{2},\ldots,i_{m}\in N}a_{ii_{2}\cdots i_{m}}x_{i_{2}}\cdots x_{i_{m}} \\ \leq& a_{is\cdots s}x_{s}^{m-1}+\sum _{(i_{2},\ldots,i_{m})\in\triangle (s; m-2)}a_{ii_{2}\cdots i_{m}}x_{s}^{m-2}x_{l}+ \cdots \\ &{}+\sum_{(i_{2},\ldots,i_{m})\in\triangle(s; k)}a_{ii_{2}\cdots i_{m}}x_{s}^{k}x_{l}^{m-k-1}+ \cdots \\ &{}+\sum_{(i_{2},\ldots,i_{m})\in\triangle(s; 1)}a_{ii_{2}\cdots i_{m}}x_{s}x_{l}^{m-2}+ \sum_{(i_{2},\ldots,i_{m})\in\triangle (s;0)}a_{ii_{2}\cdots i_{m}}x_{l}^{m-1} \\ =&a_{is\cdots s}\bigl(x_{s}^{m-1}-x_{l}^{m-1} \bigr)+\sum_{(i_{2},\ldots ,i_{m})\in\triangle(s; m-2)}a_{ii_{2}\cdots i_{m}}\bigl(x_{s}^{m-2}x_{l}-x_{l}^{m-1} \bigr) \\ &{}+\cdots+\sum_{(i_{2},\ldots,i_{m})\in\triangle(s; k)}a_{ii_{2}\cdots i_{m}} \bigl(x_{s}^{k}x_{l}^{m-k-1}-x_{l}^{m-1} \bigr)+\cdots \\ &{}+\sum_{(i_{2},\ldots,i_{m})\in\triangle(s; 1)}a_{ii_{2}\cdots i_{m}}\bigl(x_{s}x_{l}^{m-2}-x_{l}^{m-1} \bigr)+r_{i}(\mathcal {A})x_{l}^{m-1} \\ \leq&\beta_{0}(\mathcal{A}) \bigl(x_{s}^{m-1}-x_{l}^{m-1} \bigr)+\binom {m-1}{1}(n-1)\beta_{1}(\mathcal{A}) \bigl(x_{s}^{m-2}x_{l}-x_{l}^{m-1} \bigr)+\cdots \\ &{}+\binom{m-1}{m-k-1}(n-1)^{m-k-1}\beta_{m-k-1}(\mathcal {A}) \bigl(x_{s}^{k}x_{l}^{m-k-1}-x_{l}^{m-1} \bigr)+\cdots \\ &{}+\binom{m-1}{m-2}(n-1)^{m-2}\beta_{m-2}(\mathcal {A}) \bigl(x_{s}x_{l}^{m-2}-x_{l}^{m-1} \bigr)+r_{i}(\mathcal {A})x_{l}^{m-1} \\ =&\sum_{k=0}^{m-2}\binom{m-1}{k}(n-1)^{k} \beta_{k}(\mathcal {A}) \bigl(x_{s}^{m-k-1}x_{l}^{k}-x_{l}^{m-1} \bigr)+r_{i}(\mathcal{A})x_{l}^{m-1}. \end{aligned}$$

Taking $i=q$ in (6), we have that

7 $$\begin{aligned} \rho(\mathcal{A})x_{q}^{m-1} \leq&\sum _{k=0}^{m-2}\binom {m-1}{k}(n-1)^{k} \beta_{k}(\mathcal{A})x_{s}^{m-k-1}x_{l}^{k} \\ &{}+ \Biggl(r-\sum_{k=0}^{m-2} \binom{m-1}{k}(n-1)^{k}\beta_{k}(\mathcal {A}) \Biggr) x_{l}^{m-1}. \end{aligned}$$

Multiplying $x_{q}^{-(m-1)}$ on both sides of (7) gives

8 $$\begin{aligned} \rho(\mathcal{A}) \leq&\sum_{k=0}^{m-2} \binom{m-1}{k}(n-1)^{k}\beta _{k}(\mathcal{A}) \frac{x_{s}^{m-k-1}x_{l}^{k}}{x_{q}^{m-1}} + \Biggl(r-\sum_{k=0}^{m-2} \binom{m-1}{k}(n-1)^{k}\beta_{k}(\mathcal {A}) \Biggr) \frac{x_{l}^{m-1}}{x_{q}^{m-1}} \\ \leq&\sum_{k=0}^{m-2}\binom{m-1}{k}(n-1)^{k} \beta_{k}(\mathcal {A})\frac{x_{s}^{m-k-1}x_{l}^{k}}{x_{s}^{m-1}} + \Biggl(r-\sum _{k=0}^{m-2}\binom{m-1}{k}(n-1)^{k} \beta_{k}(\mathcal {A}) \Biggr) \frac{x_{l}^{m-1}}{x_{s}^{m-1}} \\ =&\sum_{k=0}^{m-2}\binom{m-1}{k}(n-1)^{k} \beta_{k}(\mathcal{A}) \biggl(\frac{x_{l}}{x_{s}} \biggr)^{k} \\ &{}+ \Biggl[r-\sum_{k=0}^{m-2}\binom{m-1}{k} (n-1)^{k}\beta_{k}(\mathcal{A}) \Biggr] \biggl( \frac{x_{l}}{x_{s}} \biggr)^{m-1}. \end{aligned}$$

Combining (5) with (8) gives

9 $$\begin{aligned} \bigl(R-\beta_{0}(\mathcal{A})\bigr) \biggl( \frac{x_{s}}{x_{l}} \biggr)^{m-1} \leq& \sum_{k=1}^{m-2} \binom{m-1}{k}(n-1)^{k}\beta_{k}(\mathcal{A}) \biggl( \frac{x_{l}}{x_{s}} \biggr)^{k} \\ &{}+ \Biggl[r-\sum _{k=0}^{m-2}\binom {m-1}{k}(n-1)^{k} \beta_{k}(\mathcal{A}) \Biggr] \biggl(\frac {x_{l}}{x_{s}} \biggr)^{m-1}. \end{aligned}$$

Multiplying $(\frac{x_{s}}{x_{l}} )^{m-1}$ on both sides of (9) gives

10 $$\begin{aligned} \bigl(R-\beta_{0}(\mathcal{A})\bigr) \biggl( \frac{x_{s}}{x_{l}} \biggr)^{2(m-1)} \leq&\sum_{k=1}^{m-2} \binom{m-1}{k}(n-1)^{k}\beta_{k}(\mathcal{A}) \biggl( \frac{x_{s}}{x_{l}} \biggr)^{m-k-1} \\ &{}+ \Biggl[r-\sum_{k=0}^{m-2} \binom{m-1}{k}(n-1)^{k}\beta_{k}(\mathcal {A}) \Biggr]. \end{aligned}$$

Note that it is not easy to get the bound of $\frac{x_{s}}{x_{l}}$ simply from (10); however, we can overcome this difficulty by using the fact that $0\leq\frac{x_{s}}{x_{l}}\leq1$ for the right-hand side of (10). Hence by (10) we have that

11 $$ \bigl(R-\beta_{0}(\mathcal{A})\bigr) \biggl( \frac{x_{s}}{x_{l}} \biggr)^{2(m-1)} \leq r-\beta_{0}(\mathcal{A}), $$

that is, $\frac{x_{s}}{x_{l}} \leq (\frac{r-\beta_{0}(\mathcal{A})}{R-\beta_{0}(\mathcal {A})} )^{\frac{1}{2(m-1)}}$, which together with (10) yields

$$\begin{aligned} \biggl(\frac{x_{s}}{x_{l}} \biggr)^{2(m-1)} \leq& \frac{\sum_{k=1}^{m-2}\binom{m-1}{k}(n-1)^{k}\beta_{k}(\mathcal{A}) (\frac {r-\beta_{0}(\mathcal{A})}{R-\beta_{0}(\mathcal{A})} )^{\frac {m-k-1}{2(m-1)}} }{R-\beta_{0}(\mathcal{A})} + \frac{r-\sum_{k=0}^{m-2}\binom {m-1}{k}(n-1)^{k}\beta_{k}(\mathcal{A})}{R-\beta_{0}(\mathcal{A})} \\ =&\zeta(\mathcal{A})^{2(m-1)}. \end{aligned}$$

The conclusion follows. □

### Theorem 6

*Let* $\mathcal{A}=(a_{i_{1}i_{2}\cdots i_{m}})\in\mathbb{R}^{[m,n]}$ *be a weakly irreducible nonnegative tensor*. *Then*

12 $$ \mathcal{L}(\mathcal{A})\leq\rho(\mathcal{A})\leq\mathcal{U}( \mathcal{A}), $$

*where*

$$\mathcal{L}(\mathcal{A})=\min_{i,j\in N} \biggl\{ a_{ij\cdots j} \biggl[\frac{1}{\zeta(\mathcal{A})^{m-1}}-1 \biggr]+r_{i}(\mathcal{A}) \biggr\} , $$

*and*

$$\mathcal{U}(\mathcal{A})=\max_{i,j\in N} \biggl\{ \sum _{(i_{2},\ldots ,i_{m})\in\bigcup_{k=1}^{m-1}\triangle(j; k)}a_{ii_{2}\cdots i_{m}} \bigl(\zeta(\mathcal{A})^{k}-1 \bigr)+r_{i}(\mathcal{A}) \biggr\} . $$

### Proof

Since $\mathcal{A}$ is a weakly irreducible nonnegative tensor, there is a Perron vector $x\in R_{++}^{n}$ such that $\mathcal{A}x^{m-1}=\rho (\mathcal{A})x^{[m-1]}$. Suppose that $x_{s}=\min_{i\in N}\{x_{i}\}$, $x_{l}=\max_{i\in N}\{x_{i}\}$. By (3) we have that for each $i\in N$,

13 $$ \rho(\mathcal{A})x_{i}^{m-1}\geq a_{il\cdots l}\bigl(x_{l}^{m-1}-x_{s}^{m-1} \bigr)+r_{i}(\mathcal{A})x_{s}^{m-1}. $$

Taking $i=s$ and multiplying $x_{s}^{-(m-1)}$ on both sides of (13), we obtain that

14 $$ \rho(\mathcal{A})\geq a_{sl\cdots l} \biggl(\frac {x_{l}^{m-1}-x_{s}^{m-1}}{x_{s}^{m-1}} \biggr)+r_{s}(\mathcal {A})=a_{sl\cdots l} \biggl[ \biggl( \frac{x_{l}}{x_{s}} \biggr)^{m-1}-1 \biggr]+r_{s}(\mathcal{A}). $$

Combining (14) with Lemma 1 gives

15 $$\begin{aligned} \rho(\mathcal{A}) \geq& a_{sl\cdots l} \biggl[ \frac{1}{\zeta(\mathcal {A})^{m-1}}-1 \biggr]+r_{s}(\mathcal{A}) \\ \geq& \min_{i,j\in N} \biggl\{ a_{ij\cdots j} \biggl[ \frac{1}{\zeta (\mathcal{A})^{m-1}}-1 \biggr]+r_{i}(\mathcal{A}) \biggr\} . \end{aligned}$$

Similarly, by the first inequality of (6), we have that for each $i\in N$,

16 $$\begin{aligned} \rho(\mathcal{A}) x_{i}^{m-1} \leq& a_{is\cdots s}x_{s}^{m-1}+\sum_{(i_{2},\ldots,i_{m})\in\triangle(s; m-2)}a_{ii_{2}\cdots i_{m}}x_{s}^{m-2}x_{l}+ \cdots \\ &{}+\sum_{(i_{2},\ldots,i_{m})\in\triangle(s; k)}a_{ii_{2}\cdots i_{m}}x_{s}^{k}x_{l}^{m-k-1}+ \cdots \\ &{}+\sum_{(i_{2},\ldots,i_{m})\in\triangle(s; 1)}a_{ii_{2}\cdots i_{m}}x_{s}x_{l}^{m-2}+ \sum_{(i_{2},\ldots,i_{m})\in\triangle (s;0)}a_{ii_{2}\cdots i_{m}}x_{l}^{m-1} \\ =& \sum_{(i_{2},\ldots,i_{m})\in\bigcup_{k=1}^{m-1}\triangle(s; k)}a_{ii_{2}\cdots i_{m}}\bigl(x_{s}^{k}x_{l}^{m-k-1}-x_{l}^{m-1} \bigr)+r_{i}(\mathcal{A})x_{l}^{m-1}. \end{aligned}$$

Taking $i=l$ and multiplying $x_{l}^{-(m-1)}$ on both sides of (16), we obtain that

17 $$\begin{aligned} \rho(\mathcal{A}) \leq& \sum_{(i_{2},\ldots,i_{m})\in\bigcup_{k=1}^{m-1}\triangle(s; k)}a_{li_{2}\cdots i_{m}} \biggl(\frac {x_{s}^{k}x_{l}^{m-k-1}-x_{l}^{m-1}}{x_{l}^{m-1}} \biggr)+r_{l}(\mathcal {A}) \\ =&\sum_{(i_{2},\ldots,i_{m})\in\bigcup_{k=1}^{m-1}\triangle(s; k)}a_{li_{2}\cdots i_{m}} \biggl[ \biggl( \frac{x_{s}}{x_{l}} \biggr)^{k}-1 \biggr]+r_{l}(\mathcal{A}). \end{aligned}$$

Combining (17) with Lemma 1 gives

$$\begin{aligned} \begin{aligned} \rho(\mathcal{A})&\leq\sum_{(i_{2},\ldots,i_{m})\in\bigcup _{k=1}^{m-1}\triangle(s; k)}a_{li_{2}\cdots i_{m}} \bigl(\zeta(\mathcal {A})^{k}-1 \bigr)+r_{l}(\mathcal{A}) \\ &\leq\max_{i,j\in N} \biggl\{ \sum_{(i_{2},\ldots,i_{m})\in\bigcup _{k=1}^{m-1}\triangle(j; k)}a_{ii_{2}\cdots i_{m}} \bigl(\zeta(\mathcal {A})^{k}-1 \bigr)+r_{i}(\mathcal{A}) \biggr\} . \end{aligned} \end{aligned}$$

The proof is completed. □

### Remark 1

It is easy to see that the bound in (12) also holds for general nonnegative tensors. In fact, if $\mathcal {A}=(a_{i_{1}i_{2}\cdots i_{m}})\in\mathbb{R}^{[m,n]}$ is a nonnegative tensor, and $\mathcal{F}=(f_{i_{1}i_{2}\cdots i_{m}})\in \mathbb{R}^{[m,n]}$ with $f_{i_{1}i_{2}\cdots i_{m}}=1$ for all $i_{r}\in N$, $r=1,2,\ldots,m$, then $\mathcal{A}+\varepsilon\mathcal {F}$ is a weakly irreducible tensor for any $\varepsilon>0$. Hence by Theorem 6 we can give the bound of $\rho(\mathcal{A}+\varepsilon \mathcal{F})$. Since the spectral radius of a nonnegative tensor is a continuous function of its entries, the bound for $\rho(\mathcal{A})$ can be obtained when $\varepsilon\rightarrow0$, which is exactly the bound in (12).

### Remark 2

Note that the first inequality of (6) can be replaced by

$$ \rho(\mathcal{A}) x_{i}^{m-1}\leq a_{is\cdots s}x_{s}^{m-1}+ \sum_{(i_{2},\ldots,i_{m})\in\triangle(s; m-2)}a_{ii_{2}\cdots i_{m}}x_{s}^{m-2}x_{l} +\sum_{(i_{2},\ldots,i_{m})\in\bigcup_{k=0}^{m-3}\triangle (s;k)}a_{ii_{2}\cdots i_{m}}x_{l}^{m-1}, $$

then, similar to the proof of Lemma 1, we can obtain that

18 $$ \frac{x_{s}}{x_{l}}\leq\delta(\mathcal{A}), $$

where

$$\delta(\mathcal{A})= \biggl\{ \frac{r-\beta_{0}(\mathcal {A})-(m-1)(n-1)\beta_{1}(\mathcal{A}) [1- (\frac{r-\beta _{0}(\mathcal{A})}{R-\beta_{0}(\mathcal{A})} )^{\frac {m-2}{2(m-1)}} ] }{R-\beta_{0}(\mathcal{A})} \biggr\} ^{\frac{1}{2(m-1)}}. $$

And hence, by the similar proof of Theorem 6, we can give another bound of spectral radius for a nonnegative weakly irreducible tensor $\mathcal{A}$ as follows.

### Corollary 1

*Let* $\mathcal{A}=(a_{i_{1}i_{2}\cdots i_{m}})\in\mathbb{R}^{[m,n]}$ *be a nonnegative weakly irreducible tensor*. *Then*

19 $$ \mathcal{L}_{1}(\mathcal{A})\leq\rho(\mathcal{A})\leq \mathcal {U}_{1}(\mathcal{A}), $$

*where*

$$\mathcal{L}_{1}(\mathcal{A})=\min_{i,j\in N} \biggl\{ a_{ij\cdots j} \biggl(\frac{1}{\delta(\mathcal{A})^{m-1}}-1 \biggr)+r_{i}( \mathcal{A}) \biggr\} , $$

*and*

$$\mathcal{U}_{1}(\mathcal{A})=\max_{i,j\in N} \biggl\{ r_{i}(\mathcal {A})-a_{ij\cdots j}\bigl(1-\delta( \mathcal{A})^{m-1}\bigr)-\sum_{(i_{2},\ldots ,i_{m})\in\triangle(j; m-2)}a_{ii_{2}\cdots i_{m}} \bigl(1-\delta(\mathcal {A})^{m-2}\bigr) \biggr\} . $$

### Remark 3

From the expression of $\mathcal{L}(\mathcal{A})$, $\mathcal {U}(\mathcal{A})$, $\mathcal{L}_{1}(\mathcal{A})$ and $\mathcal {U}_{1}(\mathcal{A})$, it can be easily obtained that

$$\mathcal{L}_{1}(\mathcal{A})\leq\mathcal{L}(\mathcal{A})\quad \text{and}\quad \mathcal{U}(\mathcal{A})\leq\mathcal{U}_{1}( \mathcal{A}). $$

Although the bound in (19) is not better than the bound in (12), it needs less computations.

Next is a comparison result for the bound in (12) and the bound in (2).

### Theorem 7

*Let* $\mathcal{A}=(a_{i_{1}i_{2}\cdots i_{m}})\in\mathbb{R}^{[m,n]}$ *be a nonnegative tensor*. *Then*

20 $$ \nu(\mathcal{A})\leq\mathcal{L}(\mathcal{A})\leq\mathcal{U}( \mathcal {A})\leq\omega(\mathcal{A}). $$

### Proof

We only prove $\nu(\mathcal{A})\leq\mathcal{L}(\mathcal{A})$, $\mathcal {U}(\mathcal{A})\leq\omega(\mathcal{A})$ can be similarly proved. Note that

$$\tau(\mathcal{A})= \biggl(\frac{r-\beta_{0}(\mathcal{A})}{R-\beta _{0}(\mathcal{A})} \biggr)^{\frac{1}{2(m-1)}}, $$

and

$$\zeta(\mathcal{A})= \biggl\{ \frac{r-\beta_{0}(\mathcal{A})-\sum_{k=1}^{m-2} [\binom{m-1}{k}(n-1)^{k}\beta_{k}(\mathcal{A}) (1- (\frac{r-\beta_{0}(\mathcal{A})}{R-\beta_{0}(\mathcal{A})} )^{\frac{m-k-1}{2(m-1)}} ) ] }{R-\beta_{0}(\mathcal{A})} \biggr\} ^{\frac{1}{2(m-1)}}. $$

Since $r\leq R$, we have

$$\sum_{k=1}^{m-2} \biggl[\binom{m-1}{k}(n-1)^{k} \beta_{k}(\mathcal {A}) \biggl(1- \biggl(\frac{r-\beta_{0}(\mathcal{A})}{ R-\beta_{0}(\mathcal{A})} \biggr)^{\frac{m-k-1}{2(m-1)}} \biggr) \biggr]\geq0. $$

Hence we can obtain $\zeta(\mathcal{A})\leq\tau(\mathcal{A})$, consequently, $\nu(\mathcal{A})\leq\mathcal{L}(\mathcal{A})$ because

$$\nu(\mathcal{A})=\min_{i,j\in N} \biggl\{ a_{ij\cdots j} \biggl( \frac {1}{\tau(\mathcal{A})^{m-1}}-1 \biggr)+r_{i}(\mathcal{A}) \biggr\} , $$

and

$$\mathcal{L}(\mathcal{A})=\min_{i,j\in N} \biggl\{ a_{ij\cdots j} \biggl[\frac{1}{\zeta(\mathcal{A})^{m-1}}-1 \biggr]+r_{i}(\mathcal{A}) \biggr\} . $$

The proof is completed. □

### Remark 4

Note that $\delta(\mathcal{A})\leq\tau(\mathcal{A})$ is also obvious. Therefore, combining the proof of Theorem 7 and Remark 3, we have that

$$\nu(\mathcal{A})\leq\mathcal{L}_{1}(\mathcal{A})\leq\mathcal{L}( \mathcal {A})\leq\mathcal{U}(\mathcal{A})\leq\mathcal{U}_{1}( \mathcal{A})\leq \omega(\mathcal{A}). $$

## Numerical examples

In this section, we use two examples to illustrate the effectiveness of our proposed results.

### Example 1

Let $\mathcal{A}=(a_{i_{1}i_{2}i_{3}i_{4}})$ be an order 4 dimension 3 tensor, where

$$\begin{aligned}& \mathcal{A}(:,:,1,1)=\left [ \textstyle\begin{array}{@{}c@{\quad}c@{\quad}c@{}} 0.6537 &0.9211 &0.4400\\ 0.9326 &0.7947 &0.2576\\ 0.1635 &0.5774 &0.7519 \end{array}\displaystyle \right ] ,\\& \mathcal{A}(:,:,2,1)=\left [ \textstyle\begin{array}{@{}c@{\quad}c@{\quad}c@{}} 0.2287 &0.6712 &0.4190\\ 0.0642 &0.7152 &0.3908\\ 0.7673 &0.6421 &0.8161 \end{array}\displaystyle \right ] , \\& \mathcal{A}(:,:,3,1)=\left [ \textstyle\begin{array}{@{}c@{\quad}c@{\quad}c@{}} 0.3174 &0.8523 &0.9509\\ 0.8145 &0.5056 &0.4440\\ 0.7891 &0.6357 &0.0600 \end{array}\displaystyle \right ] ,\\& \mathcal{A}(:,:,1,2)=\left [ \textstyle\begin{array}{@{}c@{\quad}c@{\quad}c@{}} 0.8667 &0.9970 &0.6050\\ 0.6312 &0.2242 &0.3872\\ 0.3551 &0.6525 &0.1422 \end{array}\displaystyle \right ] , \\& \mathcal{A}(:,:,2,2)=\left [ \textstyle\begin{array}{@{}c@{\quad}c@{\quad}c@{}} 0.0251 &0.7258 &0.7342\\ 0.4211 &0.3704 &0.5710\\ 0.1841 &0.8416 &0.1769 \end{array}\displaystyle \right ] ,\\& \mathcal{A}(:,:,3,2)=\left [ \textstyle\begin{array}{@{}c@{\quad}c@{\quad}c@{}} 0.9574 &0.2238 &0.6401\\ 0.2653 &0.3736 &0.1806\\ 0.9246 &0.0875 &0.0451 \end{array}\displaystyle \right ] , \\& \mathcal{A}(:,:,1,3)=\left [ \textstyle\begin{array}{@{}c@{\quad}c@{\quad}c@{}} 0.7232 &0.3839 &0.9106\\ 0.3474 &0.6273 &0.8006\\ 0.6606 &0.0216 &0.7458 \end{array}\displaystyle \right ] ,\\& \mathcal{A}(:,:,2,3)=\left [ \textstyle\begin{array}{@{}c@{\quad}c@{\quad}c@{}} 0.8131 &0.5755 &0.2486\\ 0.3833 &0.5301 &0.4516\\ 0.6173 &0.2751 &0.2277 \end{array}\displaystyle \right ] , \\& \mathcal{A}(:,:,3,3)=\left [ \textstyle\begin{array}{@{}c@{\quad}c@{\quad}c@{}} 0.8044 &0.5357 &0.9891\\ 0.9861 &0.0871 &0.0669\\ 0.0300 &0.8021 &0.9394 \end{array}\displaystyle \right ] . \end{aligned}$$

By the bound in (1), we have

$$12.624199999999998 \leq\rho(\mathcal{A})\leq17.213500000000000. $$

By the bound in (2), we have

$$12.635474838218220\leq\rho(\mathcal{A})\leq17.119219513960772. $$

By the bound in (19), we have

$$12.635521111779175\leq\rho(\mathcal{A})\leq16.789066893939726. $$

By the bound in (12), we have

$$12.635562034673926\leq\rho(\mathcal{A})\leq16.407092601408745. $$

In fact, $\rho(\mathcal{A})\approx14.265484618202352$.

And the relative errors for the exact value and the lower and upper bound of (1) respectively are $\frac{\rho(\mathcal {A})-r}{\rho(\mathcal{A})}=0.115052846932948$, and $\frac{R-\rho (\mathcal{A})}{\rho(\mathcal{A})}=0.206653714240879$.

The relative errors for the exact value and the lower bound of (2) respectively are $\frac{\rho(\mathcal{A})-\nu(\mathcal {A})}{\rho(\mathcal{A})}=0.114262489050269$ and $\frac{\omega(\mathcal {A})-\rho(\mathcal{A})}{\rho(\mathcal{A})}=0.200044721377158$.

The relative errors for the exact value and the lower and upper bound of (19) respectively are $\frac{\rho(\mathcal {A})-\mathcal{L}_{1}(\mathcal{A})}{\rho(\mathcal {A})}=0.114259245307614$ and $\frac{\mathcal{U}_{1}(\mathcal{A})-\rho (\mathcal{A})}{\rho(\mathcal{A})}=0.176901265065847$.

The relative errors for the exact value and the lower and upper bound of (12) respectively are $\frac{\rho(\mathcal {A})-\mathcal{L}(\mathcal{A})}{\rho(\mathcal{A})}=0.114256376642732$ and $\frac{\mathcal{U}(\mathcal{A})-\rho(\mathcal{A})}{\rho(\mathcal {A})}=0.150125147551858$.

This example shows that the bound in (12) is better.

### Example 2

Consider the ninth tensor generated by the MATLAB code

$$k=20; \quad \mathcal{A}=\operatorname{rand}(k,k,k,k). $$

By the bound in (1), we have

$$3.945382368958229\mathrm{e} {+}03\leq\rho(\mathcal{A})\leq 4.048737750258768 \mathrm{e} {+}03. $$

By the bound in (2), we have

$$3.945382847292296\mathrm{e} {+}03\leq\rho(\mathcal{A})\leq4.048737657610057 \mathrm{e} {+}03. $$

By the bound in (19), we have

$$3.945382847292452\mathrm{e} {+}03\leq\rho(\mathcal{A})\leq4.048519360105104 \mathrm{e} {+}03. $$

By the bound in (12), we have

$$3.945382847292460\mathrm{e} {+}03\leq\rho(\mathcal{A})\leq4.046170799928120 \mathrm{e} {+}03. $$

In fact, $\rho(\mathcal{A})\approx3.997739793470586\mathrm{e}{+}03$.

And the relative errors for the exact value and the lower and upper bound of (1) respectively are $\frac{\rho(\mathcal {A})-r}{\rho(\mathcal{A})}=0.013096756471712$ and $\frac{R-\rho(\mathcal {A})}{\rho(\mathcal{A})}=0.012756697389729$.

The relative errors for the exact value and the lower bound of (2) respectively are $\frac{\rho(\mathcal{A})-\nu(\mathcal {A})}{\rho(\mathcal{A})}=0.013096636820587$ and $\frac{\omega(\mathcal {A})-\rho(\mathcal{A})}{\rho(\mathcal{A})}=0.012756674214456$.

The relative errors for the exact value and the lower and upper bound of (19) respectively are $\frac{\rho(\mathcal {A})-\mathcal{L}_{1}(\mathcal{A})}{\rho(\mathcal {A})}=0.013096636820547$ and $\frac{\mathcal{U}_{1}(\mathcal{A})-\rho (\mathcal{A})}{\rho(\mathcal{A})}=0.012702068983443$.

The relative errors for the exact value and the lower and upper bound of (12) respectively are $\frac{\rho(\mathcal {A})-\mathcal{L}(\mathcal{A})}{\rho(\mathcal{A})}=0.013096636820545$ and $\frac{\mathcal{U}(\mathcal{A})-\rho(\mathcal{A})}{\rho(\mathcal {A})}=0.012114596987186$.

This example shows that the bound in (12) is better.

## Results and discussion

The main result of this paper is Theorem 6. From Remark 2 and the proof of Lemma 1, it is not difficult to see that the right expressions of last inequality (6) can also be replaced by many similar expressions according to the extent of magnifying inequality. Therefore, we can also obtain other bounds for the spectral radius of a nonnegative tensor. Furthermore, we notice that the bound in (12) is the best of them as the last inequality (6) reaches the optimum for all those possible expressions, which can be shown by two numerical examples above.

## Conclusions

In this paper, we propose a new bound for the spectral radius of a nonnegative tensor by estimating the ratio of the smallest component and the largest component of a Perron vector. And we prove that the proposed result improves the bound in [1].
